# Microenvironment-Responsive Hydrogels for Burn Wound Healing: From Molecular Design to Therapeutic Applications

**DOI:** 10.3390/polym18212616

**Published:** 2026-10-22

**Authors:** Qingxu Yang, Zhengxian Li, Junjie Fang, Hai He, Jiayinaer Baishan, Mengye Zhan, Yufei Yang, Pen Jin, Zhenbin Chen, Zifan Wang

**Affiliations:** 1 Key Laboratory of Biotechnology and Bioengineering of State Ethnic Affairs Commission, Biomedical Research Center, Northwest Minzu University, Lanzhou 730030, China; 2 Engineering Research Center of Key Technology and Industrialization of Cell-Based Vaccine, Ministry of Education, School of Bioengineering, Northwest Minzu University, Lanzhou 730030, China; 3 School of Chemical Engineering, Northwest Minzu University, Lanzhou 730030, China; 4 State Key Laboratory of Advanced Progressing and Recycling of Nonferrous Metal Materials, College of Materials Science and Engineering, Lanzhou University of Technology, Lanzhou 730030, China

**Keywords:** burn wound, microenvironment-responsive hydrogel, stimulus responsiveness, dynamic network, on-demand therapy

## Abstract

Burn wounds exhibit a dynamic and heterogeneous microenvironment characterized by altered pH, excessive reactive oxygen species (ROS), dysregulated protease activity, and infection-associated signals, providing potential triggers for stimuli-responsive hydrogels. However, in vitro responsiveness does not necessarily indicate effective activation under actual burn conditions, and therapeutic efficacy in burn models alone cannot confirm in situ triggering. This review therefore examines burn-responsive hydrogels through the framework of microenvironmental signal–material response–network transformation–therapeutic output. We discuss endogenous signals, including pH, ROS, and matrix metalloproteinases, together with externally controllable stimuli such as temperature, near-infrared light, and ultrasound. Molecular functionalization, dynamic crosslinking, and multi-network architectures are analyzed in relation to wound adaptability and therapeutic outputs, including antibacterial activity, oxidative-stress regulation, and tissue regeneration. Particular emphasis is placed on distinguishing material-level responsiveness, therapeutic efficacy in burn models, and in situ activation within the burn microenvironment. Future studies should quantitatively match pathological signal ranges with material activation thresholds while balancing response fidelity, wound adaptability, biosafety, manufacturability, and long-term therapeutic efficacy to facilitate clinical translation.

## 1. Introduction

The skin is the largest organ of the human body and serves not only as the first line of defense against external mechanical, chemical, and microbial insults, but also as an essential regulator of water homeostasis, immune responses, and body temperature [1]. Burns disrupt this complex barrier and are caused primarily by thermal injury, although chemical, electrical, and radiation-induced injuries also occur. Depending on injury severity, tissue damage may range from superficial epidermal injury to full-thickness destruction involving the dermis and subcutaneous tissues [2,3]. According to the World Health Organization, burns account for more than 180,000 deaths worldwide each year, with the majority occurring in resource-limited settings. In addition, many survivors require prolonged treatment and may experience scarring, functional impairment, and reduced quality of life [4]. Beyond local tissue damage, severe burns can trigger systemic inflammatory responses, metabolic disturbances, and remote organ dysfunction, further complicating clinical management [5].

Cutaneous wound healing is a highly dynamic and multifactorial process that generally proceeds through four overlapping phases: hemostasis, inflammation, proliferation, and tissue remodeling. Throughout these phases, immune cells, fibroblasts, keratinocytes, and endothelial cells coordinate tissue regeneration within the extracellular matrix (ECM) under the concerted regulation of multiple cytokines and growth factors [6,7,8]. This general four-stage framework is also adopted in reviews on wound-healing hydrogels [6,7,8]. However, the pathological environment of burn wounds can disrupt this tightly regulated repair process. Deep burns generate distinct zones of tissue injury, including zones of coagulation and stasis, and persistent inflammation, ischemia, and impaired local microcirculation may drive wound conversion, whereby initially viable tissue progresses toward necrosis [9]. Meanwhile, disruption of the skin barrier, sustained inflammatory activation, and extensive exudation collectively reshape the wound microenvironment, resulting in abnormalities such as fluctuations in pH [10,11,12,13], accumulation of reactive oxygen species (ROS) [14,15], and increased proteolytic enzyme activity [16]. These alterations are associated with an increased risk of infection and delayed repair, although the direction and magnitude of individual signals vary with wound status. Thus, a burn wound should not be regarded as a static site of tissue damage, but rather as a dynamic biochemical microenvironment that continuously evolves throughout the healing process.

Current clinical management of burn wounds still relies substantially on antimicrobial agents, conventional dressings, and artificial wound coverings. Conventional topical antimicrobials, such as silver sulfadiazine, can reduce the risk of infection; however, prolonged application may adversely affect reparative cells and interfere with re-epithelialization [17,18]. In addition, conventional dressings primarily provide passive barrier protection, while frequent dressing changes may damage newly formed tissue and exacerbate patient discomfort [19]. More importantly, the composition and function of most conventional therapeutic materials remain relatively fixed and therefore cannot readily accommodate the changing physiological requirements of a wound as it progresses from inflammation through proliferation to remodeling. Consequently, the development of responsive dressings capable of adapting their material behavior and therapeutic output to changes in wound status has emerged as an important direction in burn wound management.

Hydrogels have attracted considerable attention for wound management because of their three-dimensional hydrophilic polymer networks, high water content, and soft, tissue-like characteristics resembling those of the native ECM. By tailoring crosslinking mechanisms, network architectures, and functional groups, hydrogels can be engineered to exhibit in situ gelation, self-healing, tissue adhesion, and the delivery of drugs, growth factors, or cells, making them versatile material platforms for burn wound repair [20,21,22,23,24,25,26]. Similar advantages—including injectability, self-healing, biocompatibility, and tunable physicochemical properties—have been emphasized in previous Polymers reviews of hydrogel wound dressings [6,7]. Nevertheless, conventional hydrogels typically operate in a relatively static therapeutic mode, in which their network structure and functionality remain largely unchanged after formation, while cargo release is predominantly governed by concentration-gradient-driven diffusion or bulk degradation. Such systems therefore have limited capacity to accommodate dynamic changes in pH, redox state, and enzymatic activity during wound healing [6,7]. Microenvironment-responsive hydrogels address this limitation by incorporating functional units that recognize endogenous signals, such as pH, ROS, and proteases, or respond to externally applied physical stimuli, including temperature, light, and ultrasound. These inputs can subsequently be translated into material-level changes, including network rearrangement, degradation, swelling, and controlled cargo release [7], thereby providing a rational route toward state-dependent therapy for burn wounds. This stimulus–material response relationship is consistent with the general framework used in previous Polymers reviews of stimuli-responsive wound-healing hydrogels [6,7].

In this review, a microenvironment-responsive hydrogel is defined as a hydrated three-dimensional polymer network in which a chemically or physically defined endogenous microenvironmental signal induces a measurable change in network structure, physicochemical properties, or therapeutic output. Relevant endogenous signals include pH, reactive oxygen species, proteases, ions, metabolites, and infection-associated factors. Temperature, near-infrared light, and ultrasound are addressed separately as externally controlled stimuli.

Multifunctionality and stimulus responsiveness are distinct but complementary properties. Here, multifunctionality denotes the integration of two or more intended material or therapeutic functions within one hydrogel, such as tissue adhesion, self-healing, antimicrobial activity, antioxidant activity, sensing, or controlled drug delivery. In contrast, responsiveness denotes a stimulus-dependent change in material behavior or therapeutic output. Thus, a multifunctional hydrogel should not be classified as responsive unless a defined endogenous or externally applied stimulus is shown to regulate its network behavior or therapeutic output; conversely, a responsive hydrogel may perform only one intended therapeutic function.

Importantly, the burn wound microenvironment exhibits pronounced temporal dynamics and spatial heterogeneity [10,14,16]. Therefore, the presence of a stimulus-responsive chemical group does not necessarily demonstrate that the material will be activated in an actual burn wound. The suitability of a responsive hydrogel should be evaluated according to three related questions: whether the target signal is present in the burn wound, whether its physiologically relevant range is sufficient to reach the activation threshold of the material, and whether the resulting material response can generate a therapeutically meaningful output. This consideration is particularly important for systems originally developed for diabetic wounds, chronic wounds, infected wounds, or other pathological models, in which the direction, magnitude, and temporal profile of the relevant stimuli may differ from those encountered in burn wounds.

Existing reviews of burn hydrogels have largely summarized wound dressings according to material type, functional category, or clinical management strategy [26,27,28,29], whereas reviews of responsive hydrogels for general wound healing have commonly organized the field according to stimulus type or material function [8,30]. Indeed, previous Polymers reviews have systematically classified responsive wound-healing hydrogels according to endogenous stimuli, including pH, ROS, enzymes, and glucose, and exogenous stimuli such as temperature, light, electric fields, and magnetic fields [6,7]. Other work has similarly emphasized how local environmental conditions can guide the design of stimuli-responsive wound dressings. Collectively, these studies provide an important foundation for the material design and functional development of hydrogel-based burn therapies. Nevertheless, systematic consideration of how the dynamic characteristics of the burn wound microenvironment—including alkaline pH, local oxidative stress, and dysregulated protease activity—correspond to specific responsive chemistries remains limited. In particular, material-level validation of stimulus responsiveness, evidence of microenvironmental modulation, and therapeutic outcomes in burn wounds do not always constitute a continuous mechanistic evidence chain.

Accordingly, this review adopts a framework linking burn microenvironmental signals, material responses, network transformations, and therapeutic outputs to examine microenvironment-responsive hydrogels for burn wound management. We first discuss the biological basis and response mechanisms associated with endogenous signals, including pH, ROS, and matrix metalloproteinases, as well as externally applied physical stimuli such as temperature, light, and ultrasound. We then compare functionalization strategies based on natural and synthetic polymers and examine how dynamic crosslinking and multi-network architectures can further regulate wound adaptability and therapeutic performance. Building on these molecular and structural design principles, we analyze increasing levels of control, ranging from single-stimulus and multistimuli responsiveness to programmed therapy and integrated sensing–therapeutic systems. Finally, current evidence gaps and future directions are discussed with particular emphasis on stimulus–response matching, evaluation methodologies, material complexity, and clinical translation, with the aim of establishing a clearer materials-oriented framework for designing responsive hydrogels tailored to the actual microenvironment of burn wounds.

### Literature Search Methodology

A focused narrative literature search was conducted in PubMed and Web of Science. The final search was performed on 29 September 2026. A representative Boolean search string was (“burn wound” OR “burn injury”) AND (“hydrogel” OR “responsive hydrogel” OR “stimuli-responsive hydrogel” OR “microenvironment-responsive hydrogel”) AND (“pH-responsive” OR “ROS-responsive” OR “enzyme-responsive” OR “thermoresponsive” OR “photothermal”) AND (“wound healing”). Publications published from January 2000 to 29 September 2026 were considered.

Screening and selection were performed in two stages. First, titles and abstracts were screened to identify studies relevant to responsive hydrogel systems and burn wound healing. Second, the full texts of potentially eligible publications were assessed according to the predefined inclusion and exclusion criteria. Studies were included when they reported the design, characterization, stimulus-responsive mechanism, therapeutic function, or burn-wound application of hydrogel systems. Studies unrelated to hydrogels, lacking a defined stimulus–response mechanism, duplicating previously identified reports, or providing insufficient methodological information were excluded. Where multiple publications described closely related material systems, priority was given to the study providing the most complete mechanistic characterization or burn-relevant therapeutic evidence.

Reference lists of relevant reviews and original research articles were also manually screened to identify additional eligible publications. Studies conducted in non-burn wound models were included only when they provided transferable mechanistic or materials-design information and were explicitly distinguished from evidence obtained in burn models. This review was designed as a focused narrative synthesis rather than a systematic review or meta-analysis.

## 2. Mechanisms Underlying Burn Wound Microenvironment Responsiveness

### 2.1. Microenvironmental Characteristics of Burn Wounds and Potential Responsive Targets

The burn wound microenvironment undergoes continuous changes in acid–base balance, redox status, protease activity, and inflammation-associated mediators, with pronounced temporal and spatial heterogeneity [10,14,16]. These alterations not only participate in burn wound pathophysiology but also provide potential endogenous triggers for responsive hydrogels. Accordingly, the rational design of such systems requires an initial assessment of the actual presence, physiologically relevant range, and evidentiary basis of each candidate signal within the burn wound microenvironment.

**Figure 1:**
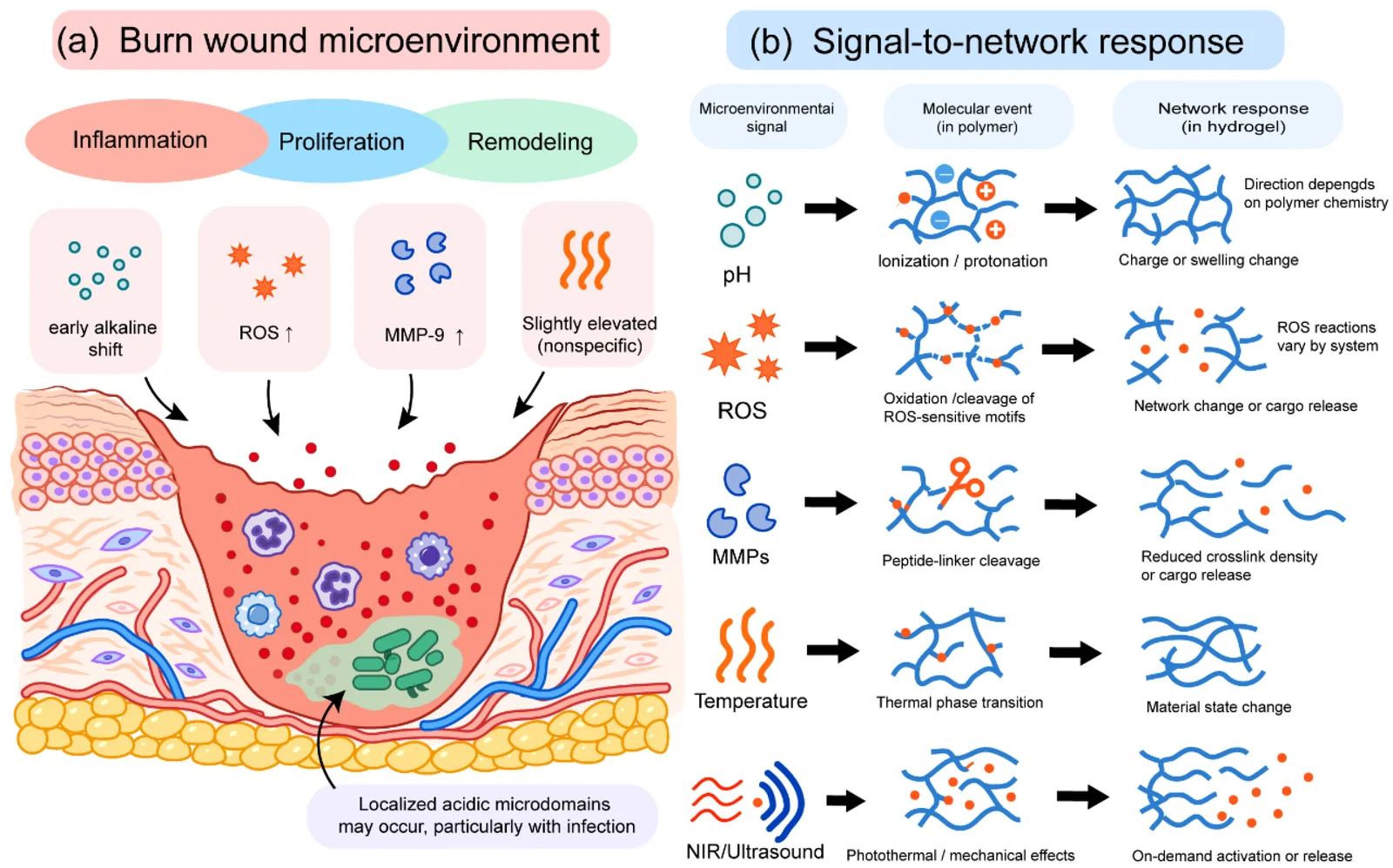
Burn wound microenvironment and signal-to-network response mechanisms of responsive hydrogels.

These dynamically evolving endogenous and exogenous cues provide the mechanistic basis for the design of microenvironment-responsive hydrogels. As summarized in Figure 1, their therapeutic relevance depends not only on the presence of a stimulus, but also on whether its magnitude and spatiotemporal profile are sufficient to trigger the corresponding molecular and network-level response.

#### 2.1.1. Endogenous Inflammation-Associated Signals

The surface of healthy skin is generally maintained in a mildly acidic state, with most studies reporting pH values of approximately 4.0–6.0 and mean values below 5.0 [28,29,30,31]. In contrast, available human evidence generally indicates a shift toward alkaline conditions in acute burn wounds. Richards and Falder [11] reported admission wound pH values ranging from 6.0 to 9.5, with a mean of 8.1, in 72 children with acute burns. Similarly, Ono et al. [13] recorded a mean wound-exudate pH of 8.55 (range, 6.5–9.0) at initial assessment in 26 patients with second-degree burns and observed that, in cases that subsequently developed local infection, an increase in pH could precede clinically apparent signs of infection. Preliminary observations by Osti et al. [32] in two patients further showed that wound pH could increase to 9.5–10.5 on day 4 after injury before gradually decreasing. Longitudinal evidence also suggests that declining pH is associated with wound improvement [10]. Taken together, these findings support considering pH in acute burn wounds as a dynamic range influenced by burn depth, infection status, time after injury, and measurement methodology, rather than as a fixed triggering threshold.

In addition to pH alterations, increased proteolytic activity after burn injury provides a potential biological basis for enzyme-responsive materials. Clinical evidence has demonstrated markedly elevated serum levels of matrix metalloproteinase-9 (MMP-9) in patients with deep partial-thickness or full-thickness thermal burns [16]; however, systemic measurements cannot be assumed to directly reflect local enzymatic activity within the wound. More direct evidence has been obtained from burn tissue and blister fluid. MMP-9 activity has been reported to be substantially higher in burned tissue than in uninjured skin [33], while alterations in MMP-8, MMP-9, and tissue inhibitor of metalloproteinases-1 (TIMP-1) have also been detected in burn blister fluid [34]. Other proteases, including neutrophil elastase, further contribute to local proteolytic activity [35]. Collectively, these observations identify proteases as potential endogenous triggers for responsive hydrogel design. Importantly, however, the presence of a systemic biomarker, increased local enzyme activity, and the availability of a specific enzyme-cleavable substrate represent distinct levels of evidence and should not be treated as interchangeable when establishing an enzyme-responsive mechanism.

#### 2.1.2. Oxidative Stress-Associated Signals

Activation of inflammatory cells, mitochondrial dysfunction, and ischemia–reperfusion after burn injury can promote the generation of ROS, including superoxide anions, hydrogen peroxide (H_2_O_2_), and hydroxyl radicals [36,37,38,39,40]. Individual ROS differ substantially in their stability, diffusion behavior, and reaction selectivity. Among them, H_2_O_2_ is relatively stable and is therefore frequently employed as a representative engineering trigger in the design and in vitro characterization of ROS-responsive materials [41,42,43,44,45]. However, direct evidence from human burns primarily supports the presence of oxidative damage rather than establishing a uniform absolute concentration of local ROS. For example, Haycock et al. [46] observed increased oxidative protein damage accompanied by reduced antioxidant capacity in blister fluid obtained from patients with superficial or partial-thickness burns.

ROS also exert context-dependent effects during wound healing. At physiological levels, ROS participate in immune-cell recruitment, pathogen clearance, angiogenesis, and cell migration, whereas persistent ROS overproduction can induce oxidative damage to lipids, proteins, and DNA and prolong inflammatory responses [14,15,46,47,48,49,50]. Consequently, the therapeutic objective of ROS regulation in burn wounds should not be reduced to indiscriminate or continuous ROS elimination, but should instead focus on restoring local redox homeostasis. Importantly, current clinical evidence remains insufficient to define an absolute local ROS concentration window in burn wounds that can be directly compared across studies. Thus, H_2_O_2_ concentrations used to demonstrate material responsiveness in vitro should not be assumed to represent the conditions encountered in human burn wounds without additional physiological validation.

#### 2.1.3. Physical and Externally Controlled Stimuli

Unlike endogenous signals such as pH, ROS, and proteases, temperature, near-infrared (NIR) light, and ultrasound primarily represent physiological or externally controllable inputs rather than burn-specific pathological biomarkers [49,51,52,53,54,55,56,57,58]. Although local wound temperature may increase in the presence of infection, most thermoresponsive material systems are designed to exploit physiological temperature or temperature changes induced by external heating or NIR irradiation, rather than relying on infection-associated temperature elevations as a robust endogenous trigger. NIR irradiation can modulate material behavior and therapeutic output through photothermal or photodynamic processes [59], whereas ultrasound can trigger material responses through mechanisms involving mechanical vibration, cavitation, piezoelectric effects, or sonodynamic processes [51,52,53,54,55]. Temperature-responsive systems, in turn, exploit polymer phase transitions to regulate hydrogel network states [56,57,58]. A defining feature of these approaches is that the timing and intensity of stimulation can be externally controlled. They should therefore be conceptually distinguished from endogenous responsive systems in which activation is governed primarily by the pathological state of the wound.

The major endogenous and externally controlled stimuli relevant to burn wound hydrogel design are summarized in Table 1.

The physiological relevance of a responsive stimulus depends on three related parameters: the presence and distribution of the stimulus in the wound, the activation threshold of the responsive chemistry, and the material output generated after activation. Therefore, a hydrogel may exhibit a clear response in vitro but remain inactive in vivo if the pathological signal does not reach the required threshold or if the response direction is incompatible with the wound condition.

Among the candidate endogenous stimuli, pH is relatively well documented in burn wounds, although it varies with burn depth, infection status, healing stage, sampling location, and measurement method. In contrast, clinical evidence for burn-associated ROS mainly demonstrates oxidative damage, while a standardized absolute concentration range for local ROS remains unavailable. Increased MMP and protease activity has also been reported in burn tissue and wound fluids, but the activity threshold required to cleave a specific responsive linker is rarely established.

### 2.2. Molecular and Chemical Basis of Microenvironment Responsiveness

Accordingly, a responsiveness claim should be supported by a defined stimulus and a measurable change in swelling, deswelling, sol–gel transition, degradation, permeability, or cargo release. Biological activity of a loaded cargo should be reported separately unless it is causally coupled to this stimulus-dependent material response [61].

Burn wounds undergo dynamic changes in pH, oxidative stress, protease activity, temperature, exudate composition, and bacterial burden [28,29,30]. However, the presence of a responsive chemical group does not by itself demonstrate burn-specific activation. Stronger evidence requires that the relevant stimulus range is measured or justified for the burn model and that the resulting change in network behavior or cargo release is experimentally verified under biologically relevant conditions.

#### 2.2.1. Molecular Mechanisms of pH-Responsiveness

pH-responsive hydrogels are commonly constructed from polymers containing weakly acidic or weakly basic groups, whose ionization states change with variations in the surrounding pH. Protonation or deprotonation alters the fixed-charge density of the polymer network and consequently affects electrostatic repulsion, osmotic pressure, hydration, swelling, mesh size, and cargo diffusion [62,63,64]. The direction, magnitude, and reversibility of these changes depend on the chemical structure, crosslinking strategy, polymer architecture, and environmental conditions [65,66,67,68].

Representative pH-responsive components include poly(acrylic acid) (PAA) and poly(methacrylic acid) (PMAA) as polyacids, as well as poly(4-vinylpyridine) (P4VP), poly(N-vinylimidazole) (PNVI), and poly(2-(dimethylamino)ethyl methacrylate) (PDMAEMA) as polybasic or weakly basic components [62,63]. These ionizable polymers can be incorporated into hydrogel networks to regulate swelling, degradation, and drug-release behavior in response to pH changes. However, the presence of an ionizable group alone does not establish burn-specific responsiveness. The practical suitability of a pH-responsive hydrogel depends on whether its ionization range, response magnitude, and response kinetics match the pH profile of the intended burn model.

The pH of burn wounds is dynamic and may vary with burn depth, healing stage, infection status, local tissue condition, and exudate composition [10,11,12,13]. Clinical observations have indicated that burn-wound pH may differ between healing and non-healing wounds and may change during the course of treatment. Therefore, acid-responsive systems, although widely investigated for chronic wounds, infected wounds, and biofilm-associated environments, should not automatically be regarded as direct physiological matches for acute burn wounds. An acid-triggered hydrogel should only be described as burn-microenvironment-responsive when the relevant acidic range and its temporal persistence have been experimentally demonstrated in the corresponding burn model [10,11,12,13].

In burn-wound applications, pH-responsive hydrogels have been explored for controlled drug release, antibacterial treatment, and promotion of tissue repair. Nevertheless, the therapeutic outcomes observed in these studies should be distinguished from evidence of burn-specific pH triggering. A hydrogel may improve wound healing through the activity of its loaded drug, antibacterial agent, antioxidant component, or adhesive property, even when pH-dependent activation has not been directly verified in the burn model. Accordingly, studies should ideally demonstrate both pH-dependent changes in hydrogel behavior or cargo release and the relevance of the selected pH range to the burn-wound microenvironment.

PDMAEMA is considered here primarily as a weakly basic, pH-responsive component because protonation of its tertiary amine groups changes the charge state and hydration of the polymer network [62,63]. Its temperature-dependent phase behavior is discussed separately in Section 2.2.4. For burn-wound applications, pH-dependent swelling or release observed in vitro should not be interpreted as burn-specific activation unless the relevant pH range, response magnitude, and temporal profile have been validated in the intended burn model [10,11,12,13].

#### 2.2.2. Molecular Mechanisms of ROS Responsiveness

ROS-responsive hydrogels rely on oxidation-sensitive chemical motifs whose oxidation changes network integrity, hydrophilicity, crosslinking density, or drug-retention capacity. Depending on the molecular design, oxidation may cleave ROS-labile linkages, convert hydrophobic groups into more hydrophilic products, or disrupt reversible crosslinks, thereby accelerating network degradation or cargo release [69]. For burn-wound applications, elevated oxidative stress alone is insufficient to establish ROS-responsive performance. The relevant evidence should link a physiologically justified ROS range to a measurable material transition or release profile and, ideally, demonstrate that this response occurs in the burn model. Systems that merely contain antioxidants should be described as ROS-scavenging or antioxidant materials unless an ROS-triggered change in the hydrogel itself has been demonstrated.

This distinction is particularly important in burn wounds. Available human evidence supports the occurrence of local oxidative damage, but remains insufficient to establish a uniform absolute ROS concentration range within the wound microenvironment. H_2_O_2_ used in material experiments therefore functions primarily as an engineering surrogate for oxidative conditions rather than as a direct quantitative representation of the human burn wound environment. Selection of ROS-sensitive chemistries should consequently be accompanied by validation that relates their activation thresholds to the oxidative conditions observed in burn-relevant models. In vitro responsiveness at a specified H_2_O_2_ concentration alone should not be considered sufficient evidence that the same response will occur in human burn wounds.

#### 2.2.3. Molecular Mechanisms of Enzyme Responsiveness

Enzyme-responsive hydrogels typically incorporate enzyme-recognizable sequences or substrates into the polymer network, thereby converting local enzymatic activity into changes in crosslinking structure [70]. For example, MMP-degradable PEG networks can be constructed using MMP-sensitive peptide sequences as cleavable crosslinks. Enzymatic cleavage of these sequences decreases the effective crosslink density, thereby promoting network degradation and/or cargo release [70].

A distinction should nevertheless be made between enzymatic degradability and MMP-specific responsiveness. Gelatin, for example, can be degraded by multiple proteolytic enzymes, including MMPs and serine proteases, whereas hyaluronic acid is predominantly degraded through hyaluronidase-mediated pathways. Enzymatic degradation of a natural polymer therefore does not, by itself, establish selective responsiveness to MMPs [70,71]. When selective MMP-triggered behavior is required, incorporating experimentally validated MMP-sensitive peptide sequences provides a more clearly defined molecular recognition mechanism [70]. For burn applications, it is additionally necessary to determine whether local activity of the target enzyme reaches the threshold required for effective substrate cleavage and to consider potential interference or nonspecific cleavage by other proteases present in the wound microenvironment.

#### 2.2.4. Molecular Mechanisms of Temperature Responsiveness

Temperature-responsive hydrogels primarily rely on temperature-dependent changes in polymer–water interactions and the hydrophilic/hydrophobic balance of polymer chains. Poly(N-isopropylacrylamide) (PNIPAM) is a representative example. When the temperature exceeds its lower critical solution temperature (LCST), dehydration of the polymer chains promotes hydrophobic association and network contraction, resulting in changes in hydrogel volume, network state, and potentially cargo-release behavior. Copolymerization and network engineering can further tune the phase-transition temperature and contraction behavior [56,58].

PNIPAM is not the only thermoresponsive polymer relevant to hydrogel design. Poly(N-vinylcaprolactam) is another representative thermoresponsive material that undergoes temperature-dependent changes in polymer–water interactions and can be incorporated into biomedical hydrogel networks [72]. PDMAEMA-containing systems may also exhibit formulation-dependent temperature transitions, including LCST- or UCST-like behavior in some polymer combinations. These transitions depend on factors such as pH, molecular weight, polymer concentration, ionic strength, solvent composition, copolymerization, and network architecture [73,74]. Because PDMAEMA also contains tertiary amine groups, pH and temperature responses may be coupled. The thermoresponsive behavior of the final hydrogel formulation should therefore be characterized experimentally rather than inferred from polymer identity alone. Relevant measurements include temperature-dependent swelling, contraction, sol–gel transition, rheological behavior, transmittance or turbidimetry, and heating–cooling reversibility.

Unlike pH, ROS, and proteases, temperature is more appropriately regarded as a physiological or externally applied physical trigger than as a burn-specific pathological signal. Accordingly, the principal value of thermoresponsive chemistry in burn wound treatment lies in enabling functions such as in situ gelation, temperature-dependent mechanical or structural regulation, and externally controlled thermal actuation, rather than specifically sensing burn pathology.

NIR light and ultrasound differ further from the four mechanisms discussed above because their effects on hydrogels are commonly mediated through physical energy conversion rather than direct chemical changes in the polymer chains. NIR-responsive systems typically incorporate photothermal agents, such as polydopamine or black phosphorus, to convert optical energy into heat, or photosensitizing components that generate ROS through photodynamic processes; these effects can subsequently regulate network properties or therapeutic outputs. Ultrasound-responsive systems can exploit cavitation, mechanical vibration, piezoelectric effects, or sonodynamic processes to trigger structural changes or cargo release. Because the design of these systems depends substantially on the incorporation of functional components and control of externally applied parameters rather than solely on intrinsic stimulus-sensitive polymer chemistry, their specific material configurations are not discussed further in this section. Instead, representative NIR- and ultrasound-responsive systems are considered in Section 3 and Section 4 in the context of their material design and therapeutic applications.

Taken together, whether the burn wound microenvironment can serve as an effective trigger for a responsive hydrogel ultimately depends on the correspondence between the pathophysiological signal window and the material response window. Endogenous signals such as pH, ROS, and proteases provide information about the pathological state of the wound, but their temporal dynamics, spatial heterogeneity, and interpatient variability make it difficult to define universal triggering thresholds. By contrast, temperature, NIR irradiation, and ultrasound provide more controllable inputs, but require physiological conditions or externally supplied energy. Thus, the objective of responsive hydrogel design should not be to maximize the number of incorporated response modalities. Rather, priority should be given to selecting target signals with a well-supported basis in burn pathophysiology and matching the response direction, activation threshold, and resulting network transformation to conditions that are realistically encountered in the wound.

### 2.3. Characterization and Validation of Stimulus Responsiveness

For pH-responsive hydrogels, swelling ratio, swelling kinetics, network charge, rheological properties, degradation behavior, and cargo release should be measured across a physiologically relevant pH range. Repeated pH cycling can be used to evaluate response reversibility and stability. Because burn wounds contain proteins, salts, and cellular debris, testing in simulated wound fluid or protein-containing media may provide more physiologically relevant information than testing only in simple buffer solutions.

For thermoresponsive hydrogels, temperature-dependent swelling, contraction, sol–gel transition, rheological behavior, transmittance or turbidimetry, and heating–cooling reversibility should be reported. The apparent transition temperature should be determined using the final hydrogel formulation rather than inferred from the bulk polymer alone.

Water contact-angle measurements at different pH or temperatures may provide complementary information about surface wettability. However, contact angle mainly reflects surface properties and should not replace bulk hydrogel characterization. Stimulus-dependent changes in drug release, degradation, adhesion, antibacterial activity, or regenerative activity should also be evaluated whenever these outputs are claimed to be responsive.

To distinguish true stimulus responsiveness from baseline material performance, the response magnitude and kinetics should be quantitatively compared under relevant stimulus and matched control conditions (Table 2).

Mechanical stability and biological performance provide supporting information but do not, by themselves, establish stimulus responsiveness. The relevant stimulus conditions, including pH, temperature, stimulus concentration, ionic strength, exposure duration, and sample geometry, should be reported where applicable.

## 3. Material Platforms and Functionalization Strategies for Responsive Hydrogels in Burn Wound Healing

The construction of responsive hydrogels depends fundamentally on whether the polymer backbone provides suitable sites for functionalization while retaining the biological and material properties required for wound treatment. Natural polymers generally exhibit favorable biocompatibility and tissue-relevant bioactivity but often have limited mechanical stability and intrinsic responsiveness. In contrast, synthetic polymers offer greater structural tunability, facilitating precise regulation of response thresholds and network properties, but generally lack the inherent biological recognition features of natural macromolecules [7,75]. Accordingly, the design priorities of these two material classes differ. Natural polymers are primarily modified to expand their responsive and network-forming capabilities while preserving their intrinsic biological properties, whereas synthetic polymers more commonly rely on engineering of monomers, side chains, and crosslinking sites to establish controllable stimulus–response relationships.

Across material classes, functionalization should be described according to the role of each component: a responsive element, a network-forming element, or a therapeutic/interfacial effector. Only the responsive element establishes a stimulus-responsive mechanism.

### 3.1. Functionalization of Natural Polymer-Based Responsive Hydrogels

Natural polymers, including polysaccharides, proteins, and their derivatives, generally exhibit favorable biocompatibility and biodegradability and contain modifiable functional groups, such as hydroxyl, carboxyl, and amino groups, that provide chemical handles for incorporating responsive moieties and constructing dynamic networks [75,76,77,78,79]. The multiple chemically reactive sites of natural polysaccharides, for example, facilitate their derivatization and functionalization. The role of chemical functional groups in regulating polysaccharide-based hydrogel formation and wound-healing performance has been discussed in previous reviews [77,78,79]. Their functionalization therefore generally follows a common design principle: preserving the biological characteristics of the natural backbone while introducing additional responsiveness or network-forming capacity through oxidation, substitution, grafting, or composite formation.

#### 3.1.1. Chitosan and Its Derivatives

Chitosan contains abundant amino and hydroxyl groups and exhibits inherent antibacterial and hemostatic properties; however, its limited solubility and insufficient stability when used as a single-component network restrict its direct application [75,76,77,78,79,80]. Functionalization strategies therefore primarily aim to improve aqueous solubility, increase the availability of reactive sites, and introduce stimulus-sensitive structures. Carboxymethylation can enhance water solubility while providing pH-dependent charge regulation, whereas quaternization can strengthen cationic antibacterial activity. Thiolation and catechol grafting expand the range of interfacial interactions and network-forming mechanisms. In addition, the amino groups of chitosan can react with aldehyde groups to form dynamic Schiff-base networks [65,80,81].

On this basis, incorporation of ROS-sensitive structures can further couple burn-associated oxidative changes to network regulation or cargo release. Yang C. et al. [82] developed a Cur@CMCTS-Tk system in which H_2_O_2_-induced cleavage of thioketal linkages promoted curcumin release, thereby establishing a direct connection between an oxidative trigger and therapeutic cargo delivery. In this system, thioketal cleavage constitutes the ROS-responsive mechanism, whereas ROS scavenging, NF-κB inhibition, and antibacterial activity represent therapeutic outputs. Similarly, the thermoresponsive chitosan/astaxanthin/ibuprofen system investigated by Yang X. et al. [83] primarily exploited temperature-induced gelation at approximately 37 °C for the treatment of electrical burns. Thus, the objective of chitosan functionalization should not simply be to maximize the number of chemical modifications, but rather to define whether each incorporated component serves as a responsive element, network-forming element, or therapeutic effector. Other multistimuli-responsive chitosan systems may accordingly provide transferable material-design principles, although their relevance to burn-specific triggering conditions requires independent evaluation [84].

#### 3.1.2. Sodium Alginate and Its Derivatives

Sodium alginate is rich in carboxyl groups and can form ionically crosslinked networks through coordination with Ca^2+^, enabling mild gelation and favorable shape adaptability [85]. However, conventional alginate networks lack intrinsic cell-adhesive motifs and active stimulus responsiveness. Oxidation, sulfation, and blending with other polymers have therefore been employed to further regulate their network characteristics and biological functions [75,85,86]. Periodate oxidation introduces aldehyde groups that can form dynamic linkages with amine-containing polymers, whereas sulfation modifies the biological regulatory properties of the alginate backbone [85,86].

These examples are better described as microenvironment-modulating rather than microenvironment-triggered systems. The sulfated alginate/Prussian blue nanozyme system developed by Huang et al. [86], for example, improved the wound microenvironment primarily through ROS scavenging and immunomodulation rather than through a demonstrated ROS-triggered network transformation. In the Zn^2+^-crosslinked alginate system reported by Niculescu et al. [87], Zn^2+^ primarily contributed to network crosslinking and antibacterial activity. Although both strategies act on biologically relevant aspects of the burn wound environment, neither should be classified as a corresponding stimulus-triggered network solely on the basis of its therapeutic interaction with the wound microenvironment.

#### 3.1.3. Hyaluronic Acid and Its Derivatives

Hyaluronic acid (HA) is highly biocompatible and participates in cell migration and tissue repair; however, native HA has limited mechanical stability and therefore generally requires additional crosslinking or functionalization [71]. Methacrylated hyaluronic acid (MeHA) can form mechanically stabilized networks through photocrosslinking, and MeHA dressings incorporating pro-angiogenic peptides have been investigated for deep partial-thickness burn repair [88]. Catechol grafting can further enhance wet-tissue adhesion, and related dual-cured HA systems have been applied to full-thickness burns [89]. These studies demonstrate the functional versatility of HA modification, but photocuring and enhanced adhesion are network-construction and interfacial functions, respectively, rather than evidence of microenvironment responsiveness.

When stimulus-sensitive components are additionally introduced, HA can participate in responsive network architectures. The HA/gelatin/nanozyme composite developed by Zheng et al. [90], for example, exhibited dual pH/ROS responsiveness together with redox regulation. However, because the acidic/high-ROS triggering conditions in that study were derived from a non-burn complex wound model, the work primarily demonstrates the transferability of the material-design strategy. Whether the same triggering conditions are reached in acute burn wounds requires independent validation. This distinction is important because stimuli-responsive polysaccharide hydrogels can indeed convert environmental signals into degradation and subsequent cargo release, but the existence of such a mechanism does not by itself establish its physiological relevance to a particular wound type.

#### 3.1.4. Gelatin and Other Natural Polymers

Gelatin retains some native cell-adhesive sequences, whereas gelatin methacryloyl (GelMA) can be photocrosslinked to form structurally stable hydrogel networks. Its stimulus responsiveness, however, generally requires the incorporation of additional functional components. For example, Fang et al. [91] constructed a boronic acid-functionalized GelMA/caffeoyl chitosan system combining stable photocrosslinking with dynamic caffeoyl/boronate interactions for the treatment of combined radiation–burn injury. In this system, photocrosslinking functions primarily as a network-construction method rather than as a burn microenvironmental stimulus. A stimulus-responsive mechanism should be claimed only when a defined pathological or externally applied signal subsequently regulates the network structure, degradation behavior, or therapeutic output.

Other natural materials frequently serve as structural reinforcements or functional components within composite networks. GelMA, for instance, can be combined with acellular dermal matrix (ADM) to construct multifunctional hydrogels for burn treatment. In the GelMA/ADM composite developed by Zhao et al. [92], quaternized antibacterial components, ROS-scavenging capacity, and immunomodulatory activity acted cooperatively to promote the repair of infected burns. These therapeutic effects arose predominantly from the intrinsic activities of the incorporated components rather than from microenvironment-triggered release or network transformation. This system therefore illustrates the potential of gelatin-based natural polymers for multifunctional integration, but its design logic should be distinguished from that of a microenvironment-responsive system.

Cellulose nanofibers can be incorporated to reinforce flexible hydrogel networks [93], while dopamine-grafted oxidized pectin has been used to improve adhesion and self-healing properties in hydrogels evaluated in burn models [94]. A carnosic acid (CA)/3-aminophenylboronic acid (APBA) hydrogel provides a more explicit example of stimulus-dependent behavior, exhibiting pH/ROS-associated release and therapeutic effects in a deep-burn model [95]. Nevertheless, in vitro demonstration of stimulus-triggered behavior and therapeutic efficacy in an animal burn model constitute different levels of evidence. The latter should not be used retrospectively to infer that ordinary acute burn wounds necessarily reach the same triggering thresholds employed during material characterization.

Overall, the functionalization of natural polymers can be summarized as a progression from preserving the native backbone, to introducing functional sites, and ultimately to establishing responsive or network-forming capabilities. Increasing the degree of chemical modification can expand the functional repertoire of natural polymers, but may simultaneously increase fabrication complexity and alter the intrinsic characteristics that initially motivated their use. The central design objective should therefore shift from maximizing functionalization toward establishing clear functional assignments among the polymer backbone, responsive moieties, network-forming units, and therapeutic components.

### 3.2. Molecular Design of Synthetic Polymer-Based Responsive Hydrogels

Synthetic polymers offer a high degree of structural tunability, allowing network behavior to be engineered through monomer composition, side-chain functionality, and crosslinking chemistry. Their major limitation is the general lack of intrinsic cell-recognition motifs and biological regulatory functions characteristic of many natural polymers [7,27]. Accordingly, in contrast to the post-synthetic functionalization commonly employed for natural polymers, synthetic systems place greater emphasis on molecular engineering to establish a direct relationship between stimulus input and material response.

#### 3.2.1. pH-Responsive Networks Based on Ionizable Side Chains

Poly(meth)acrylate-based materials can exploit the protonation and deprotonation of pendant carboxyl or amino groups to alter network charge and thereby generate pH-dependent structural changes. Poly(2-(dimethylamino)ethyl methacrylate) (PDMAEMA) is primarily recognized as a pH-responsive polymer because protonation of its tertiary amine groups alters the charge state and hydration of the polymer chains. Depending on molecular weight, concentration, solvent composition, copolymerization, and network architecture, PDMAEMA-containing systems may also exhibit temperature-dependent phase behavior, including LCST- or UCST-like transitions. In some composite systems, an electric-field response may arise from conductive or electroactive components and should not be attributed to PDMAEMA alone without direct evidence. A composite system combining PDMAEMA with modified chitosan has demonstrated pH-responsiveness together with system-dependent temperature behavior and controlled papain release [74]. However, the available evidence for this system is primarily based on material characterization and in vitro evaluation, without validation in an animal burn model.

For such materials, the critical consideration is whether the direction and operating window of the response are compatible with the burn wound microenvironment. Some tertiary amine-containing systems undergo their most pronounced changes under acidic conditions, whereas available evidence for acute burn wounds generally indicates a shift toward alkaline conditions. Their transfer from other disease settings to burn treatment therefore requires recalibration of the relevant response window. Acid-responsive peptide–polymer systems and pH-triggered antibiotic-delivery platforms developed for other pathological conditions [96,97] may provide useful molecular-design principles, but should not be regarded as direct evidence of burn-specific responsiveness. Similarly, although Carbomer 940 has been investigated for burn treatment [98], the reported study itself does not establish a pH-triggered mechanism. Thus, the presence of ionizable groups within a polymer is insufficient to establish physiologically relevant pH-responsiveness unless the corresponding material transition is demonstrated within a burn-relevant pH range.

#### 3.2.2. Thermoresponsive Networks Based on Polymer Phase Transitions

Poly(N-isopropylacrylamide) (PNIPAM) is a representative thermoresponsive polymer in which changes in polymer-chain hydration near the lower critical solution temperature (LCST) can regulate hydrogel volume and network behavior [56,57,58]. Copolymerization and network engineering can further tune the transition temperature, extent of contraction, and structural stability of PNIPAM-based systems [56,57,58].

In multicomponent systems, however, the component responsible for each responsive behavior should be explicitly identified. In the silk fibroin/alginate nanoparticle–PNIPAM system developed by Rezaei et al. [99], pH-dependent behavior was primarily associated with the drug-loaded nanoparticles, whereas PNIPAM functioned as the thermoresponsive matrix. In the quaternized chitosan/NIPAM system investigated by Deng et al. [100], PNIPAM provided temperature responsiveness, whereas the antibacterial activity originated from quaternized chitosan. These examples illustrate that the coexistence of multiple functional components does not necessarily mean that the system responds to an equivalent number of independent stimuli. Accordingly, the thermoresponsive function of PNIPAM should be distinguished from the intrinsic antibacterial activity provided by quaternized chitosan.

#### 3.2.3. PEG Responsive Networks Engineered Through End Groups and Crosslinking Sites

Polyethylene glycol (PEG) lacks pronounced intrinsic stimulus responsiveness, but its network behavior can be programmed through the design of functional end groups, linkers, and crosslinking sites. For example, functionalized PEG can be crosslinked with MMP-sensitive peptides to generate enzyme-responsive networks in which proteolytic activity induces network degradation and/or cargo release [70,101]. ROS-cleavable poly(thioketal) linkers can likewise be incorporated to construct oxidation-responsive degradable networks [102]. However, much of the evidence supporting these strategies derives from tissue-engineering or non-burn models. Their application to burn wounds therefore requires independent confirmation that the relevant local signals reach levels sufficient to activate the designed chemistry.

Cleavable linkages such as disulfide bonds can also regulate PEG-network stability and macromolecular release [103], but their triggering chemistry warrants particular attention in the context of burns. Disulfide cleavage is commonly promoted under strongly reducing conditions, such as those associated with millimolar concentrations of glutathione, whereas burn wounds are characterized by oxidative stress associated with excessive ROS production [14]. The nominal pathological direction of these two conditions is therefore different. Consequently, disulfide-based reduction-responsive systems should not automatically be assumed to undergo efficient cleavage in the burn wound microenvironment, and their applicability requires careful evaluation under burn-relevant redox conditions. Overall, the principal advantage of PEG lies in the high design flexibility of its crosslinking sites and responsive linkers, whereas its relevance to burn treatment ultimately depends on matching the selected chemistry to the actual signals present within the wound.

#### 3.2.4. PVA and Other Functional Synthetic Networks

Poly(vinyl alcohol) (PVA) contains abundant hydroxyl groups and can form hydrogel networks through physical crosslinking or dynamic interactions, while also allowing integration with boronic acid-containing moieties, polyphenols, and functional nanomaterials. Some PVA-based composite systems have extended stimulus responsiveness beyond drug delivery toward visualization of wound status. For example, the PVA/berberine/aggregation-induced emission (AIE) hydrogel reported by Fang et al. [104] enabled pH visualization and was evaluated in a burn model. Sang et al. [105] developed a GPC-BTC system exhibiting pH-dependent functional changes together with NIR-mediated photothermal antibacterial activity for full-thickness burn treatment.

Other composite systems developed in non-burn wound models have incorporated functions such as temperature visualization, electrical conductivity, or photothermal therapy [106,107], providing potentially transferable design strategies for burn hydrogels. Nevertheless, these functions should be classified according to their actual sensing and triggering mechanisms. For each claimed responsive pathway, the stimulus, responsive element, and resulting material output should be explicitly identified and connected through a defined causal sequence: stimulus → responsive element → material output.

Overall, synthetic polymer design follows a molecular-engineering pathway of functional-unit incorporation → regulation of stimulus responsiveness → network-level output. Compared with natural polymers, synthetic systems provide greater freedom to engineer responsive structures and tune their activation characteristics, but their limited intrinsic bioactivity often necessitates supplementation with biologically functional components. Hybridization of natural and synthetic polymers should therefore not be regarded simply as increasing compositional complexity. Rather, it can provide a rational division of functions, in which natural components contribute biological interactions while synthetic components provide structural control and programmable responsiveness.

Representative responsive hydrogel systems and their reported responses in burn-wound models are summarized in Table 3.

## 4. Structural Design and Functional Optimization of Microenvironment-Responsive Hydrogels

The presence of responsive moieties alone does not ensure that a hydrogel will generate an effective output in the burn wound environment. Pathological or externally applied stimuli must be further transduced into changes in crosslinking sites, network architecture, or cargo state that ultimately produce measurable material behaviors and therapeutic effects. Accordingly, this section discusses responsive hydrogel design across four sequential levels: network response, wound adaptation, therapeutic output, and therapeutic control.

### 4.1. Molecular Design and Structural Regulation of Responsive Networks

The design of a responsive network is governed primarily by its triggering conditions, the kinetics of network transformation, and the reversibility of the response. From the perspectives of network connectivity and structural hierarchy, regulation can be broadly achieved through noncovalent interactions, dynamic covalent linkages, and cooperative multi-network architectures. Noncovalent interactions enable rapid and reversible network rearrangement; dynamic covalent bonds provide a balance between structural connectivity and bond exchange; and multi-network architectures allow different networks to assume distinct functions, thereby coordinating dynamic responsiveness with structural stability.

#### 4.1.1. Dynamic Networks Mediated by Noncovalent Interactions

Hydrogen bonding, electrostatic interactions, metal coordination, and host–guest interactions can enable network rearrangement through continuous dissociation and reassociation, with their dynamic behavior governed primarily by interaction strength and exchange kinetics [112]. Catechol–Fe^3+^ and other polyphenol–metal systems can regulate network stability through coordination interactions [105,112], whereas the interaction strength of polyphenol-based hydrogen-bonded networks can be tuned by varying component content and grafting density [113]. Components such as cellulose nanofibers can additionally provide structural reinforcement through physical entanglement [93].

A central design trade-off in noncovalent networks lies between dynamic rearrangement and structural retention. Interactions that are too weak may be insufficient to maintain network integrity, whereas excessively strong interactions can restrict chain mobility and impede network reorganization. The objective is therefore not to maximize dynamic exchange, but to establish an appropriate balance between the rate of structural rearrangement required for the intended function and the stability necessary to maintain the hydrogel network.

#### 4.1.2. Responsive Networks Constructed Through Dynamic Covalent Bonds

Dynamic covalent structures, including imine, acylhydrazone, boronate ester, and disulfide linkages, can undergo bond exchange or cleavage while retaining the mechanical and structural advantages associated with covalent connectivity, thereby imparting a degree of network adaptability [67,114,115]. Their macroscopic behavior depends on the type of dynamic bond, crosslinking density, and the location of the responsive linkage within the network architecture. When stimulus-sensitive bonds serve directly as major crosslinking points, changes in these bonds can be more effectively translated into network degradation, structural rearrangement, or cargo release. The general design principles of pH-responsive C=N linkages and dynamic covalent hydrogels for wound repair have been summarized in previous studies [114,115].

ROS-sensitive linkages provide a representative example of this design principle. Positioning oxidizable or ROS-cleavable structures at network crosslinking sites can translate changes in oxidative conditions into alterations in network integrity and/or cargo release [41,116,117]. A dynamic CMCS/OAlg network has similarly been reported to regulate exosome release through ROS-associated network changes [118]. However, because this evidence was obtained in a diabetic wound model, it should be regarded as a transferable network-design strategy rather than direct validation of responsiveness in burn wounds.

For burn applications, the key consideration remains whether the chemical response window of a dynamic covalent bond corresponds to the actual stimulus conditions within the wound. Some acid-sensitive C=N-based systems were originally developed for acidic chronic wounds or other pathological environments, whereas acute burn wounds generally exhibit a tendency toward alkaline conditions. Their triggering conditions therefore cannot be directly extrapolated to burns [119]. Rational network design should simultaneously consider the direction of the response, activation threshold, and bond-exchange kinetics, rather than simply maximizing stimulus sensitivity.

#### 4.1.3. Functional Division Within Multi-Network Architectures

A single network is often required to provide both dynamic responsiveness and structural support, potentially creating a trade-off between adaptability and mechanical stability. Multi-network architectures offer an alternative strategy by assigning distinct functions to different structural components. A relatively stable network can preserve overall structural integrity, whereas a dynamic network can mediate structural rearrangement, energy dissipation, or functional output. Such functional division can partially decouple network stability from dynamic responsiveness.

For example, Schiff-base linkages and catechol–Fe^3+^ coordination can be combined to construct a dual-crosslinked network [120]. Dual crosslinking should therefore be interpreted as a network-construction strategy rather than as evidence of dual-stimulus responsiveness, unless two defined stimuli independently regulate the material output. A CMCS/puerarin double-network hydrogel further exhibited pH-dependent release under mildly alkaline conditions and demonstrated antibacterial and oxidative-stress-regulating effects in an infected burn model [108]. This example illustrates that a multi-network architecture does not inherently confer responsiveness; rather, responsiveness depends on the presence of a defined process through which a stimulus is transduced into a network-level or therapeutic output. The chitosan-based composite burn hydrogel developed by Huo et al. [121] represents another type of dynamic injectable system, and its network architecture and functional mechanisms should therefore be described independently rather than being incorporated into the same dual-stimulus framework.

### 4.2. Design of Wound-Adaptive Properties

Responsive networks can function effectively only when they maintain sufficient contact with the wound surface and preserve structural integrity during treatment. Tissue movement, persistent moisture, localized mechanical damage, and irregular wound geometry impose distinct requirements on the mechanical adaptability, wet-tissue adhesion, self-healing capacity, and in situ gelation of hydrogels used for burn wounds. The objective of engineering these properties is not to maximize individual performance metrics, but to maintain a stable and functional material state under realistic wound conditions. Because wound-adaptive properties and stimulus responsiveness often share molecular mechanisms, particularly dynamic crosslinking, network design should consider responsive behavior and wound adaptability as interconnected properties rather than optimizing them independently.

#### 4.2.1. Network Toughness and Mechanical Compatibility with Tissue

Burn wound dressings should maintain continuous coverage during tensile, compressive, and bending deformation while avoiding excessive stiffness that could restrict tissue movement. Mechanical design therefore requires a balance between structural retention and deformability. Dual or multiple crosslinking strategies can distribute mechanical loads among different network interactions. For example, a dual-crosslinked system based on Schiff-base bonds and catechol–Fe^3+^ coordination can improve mechanical stability while retaining dynamic network rearrangement [120]. Cellulose nanofibers can reinforce flexible networks through physical entanglement [93], whereas graphene-based supramolecular systems can further contribute to energy dissipation [122]. Thus, mechanical optimization of burn hydrogels should not be reduced to maximizing strength or modulus; rather, the material should preserve network continuity and functional integrity during repeated deformation.

#### 4.2.2. Wet-Tissue Interfacial Adhesion

Wound exudate, blood, and proteins can interfere with direct contact between a hydrogel and injured tissue, creating a need for stable yet appropriately controlled adhesion under wet conditions [123]. Catechol groups can enhance wet-tissue adhesion through hydrogen bonding, metal coordination, and oxidative coupling [123], and related HA-based and catechol–metal systems have been employed to improve interfacial bonding [60,89]. Aldehyde-functionalized polysaccharides can additionally form in situ linkages with amino groups on tissue surfaces through Schiff-base chemistry.

Stronger adhesion, however, is not necessarily therapeutically preferable. Insufficient interfacial bonding can lead to hydrogel displacement or detachment, whereas excessive adhesion may increase disruption of newly formed tissue during dressing removal. The relevant design objective is therefore to balance stable fixation during treatment with low-trauma removal when replacement is required.

#### 4.2.3. Self-Healing Mediated by Dynamic Networks

Localized network damage caused by tissue movement can disrupt the continuous coverage provided by a hydrogel. Reversible crosslinking provides a molecular basis for reconnection after such damage; however, the mere presence of dynamic bonds does not demonstrate effective self-healing. Functionally relevant self-healing requires the damaged network to recover the structural or functional properties necessary for continued operation.

Dynamic boronate ester linkages in PVA-based networks can promote reconnection after disruption, while Schiff-base networks can restore interfacial continuity through bond exchange. Polyphenol-mediated hydrogen bonding and catechol-associated interactions can likewise contribute to network recovery [94,124,125]. As with other dynamic network properties, self-healing requires a balance between bond-exchange capacity and initial structural stability. Dynamic linkages are therefore frequently combined with reinforcing structures to achieve both recoverability and sufficient network integrity. This structural rationale is consistent with previous hydrogel-dressing literature in which dynamic crosslinking is used to impart self-healing and injectable properties.

#### 4.2.4. In Situ Gelation for Adaptation to Irregular Wounds

In situ gelation enables a flowable precursor to first conform to the wound geometry and subsequently form a stable network, thereby facilitating coverage of irregular tissue defects [120,126]. The key requirement is not injectability alone, but the coordinated sequence of precursor flow, wound filling, and network fixation within an appropriate time window.

Schiff-base formation and metal coordination can facilitate rapid network formation, with some dual-crosslinked systems achieving gelation within approximately 1 min [120]. HA/gelatin, chitosan/dextran, and other injectable systems have likewise demonstrated the potential for in situ gelation [69,83,90,126]. Faster gelation, however, does not necessarily provide better wound adaptation. Excessively rapid network formation may shorten the time available for spreading, conformational filling, and clinical manipulation, whereas slow gelation may permit precursor migration away from the target site. Gelation kinetics should therefore be matched to the practical requirements of wound filling and material placement.

Overall, wound adaptability does not depend on maximizing any single material property. Rather, an effective hydrogel should preserve structural integrity during tissue deformation, maintain controlled adhesion at wet interfaces, re-establish network continuity after localized damage, and conform to irregular wound geometries through appropriately timed gelation.

**Figure 2:**
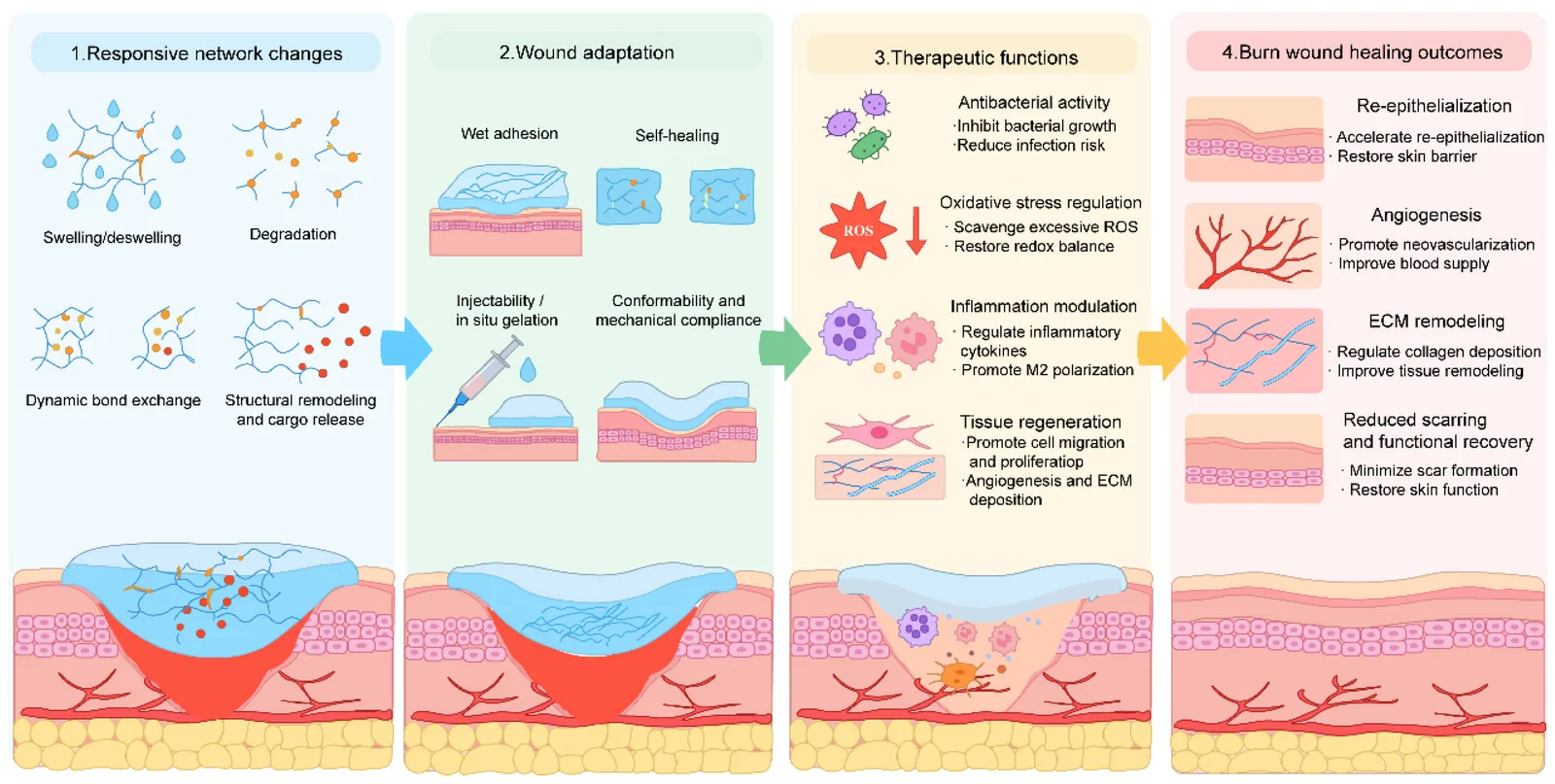
Translation of hydrogel network responses into wound adaptation, therapeutic functions, and burn wound healing outcomes.

The therapeutic value of a responsive hydrogel ultimately depends on whether stimulus-induced network changes can be translated into stable wound adaptation and biologically meaningful outputs. Figure 2 summarizes this progression from network-level responses to wound adaptation, therapeutic functions, and healing outcomes.

### 4.3. Construction of Multifunctional Therapeutic Systems

Multifunctional therapeutic systems integrate more than one therapeutic function. Their value is enhanced when an independently verified material response regulates at least one of these functions. Stimulus–therapy coupling is established when a pathological or externally applied signal changes the magnitude, timing, localization, or mode of delivery of a therapeutic output.

#### 4.3.1. Inflammatory Phase: Infection Control and Regulation of Oxidative Stress

During the early stage of burn wound healing, infection control and mitigation of excessive oxidative damage represent major therapeutic requirements. Antibacterial activity can arise intrinsically from functional components such as quaternized chitosan and Zn^2+^/Cu^2+^ [87,100,127], or can be coupled to stimulus-responsive delivery. For example, infection-associated pH changes have been exploited to promote on-demand release of TOB, with the strategy validated in a *P. aeruginosa*-infected burn model [109]. Multilevel pH-responsive systems have likewise been investigated for the treatment of infected burns [128]. Janus architectures can spatially segregate functions such as unilateral antibacterial activity and hemostasis and therefore provide a transferable structural concept from non-burn wound dressings [129]. Hydrogel-mediated delivery of nitric oxide (NO) can additionally combine local antibacterial effects with regenerative functions such as promotion of angiogenesis [130]; however, NO delivery itself represents an active-molecule delivery strategy rather than evidence of stimulus responsiveness.

Externally applied photothermal therapy provides another route for infection control. Materials such as polydopamine and black phosphorus can convert NIR irradiation into localized thermal effects [60,105,107,131]. In parallel, polyphenols, ROS-sensitive linkages, and nanozymes can contribute to the regulation of oxidative stress [82,86,132,133,134]. Direct ROS scavenging should therefore be distinguished from ROS-triggered material degradation, network rearrangement, or cargo release. The therapeutic objective during the inflammatory phase should therefore not be simply to maximize antibacterial activity or ROS elimination, but to coordinate pathogen control, redox homeostasis, and protection of host tissue.

#### 4.3.2. Proliferative Phase: Regenerative Support and Bioactive Delivery

As excessive inflammation and infection are brought under control, therapeutic priorities shift toward re-epithelialization, angiogenesis, and cell migration. Natural polymers such as gelatin can provide cell-interactive matrices, while localized delivery of bioactive molecules can further support tissue regeneration.

MMP-sensitive PEG networks provide a mechanism for coupling local proteolytic activity to network degradation and/or therapeutic cargo release [70,101]. However, current evidence for these systems derives predominantly from tissue-engineering or non-burn models. Although abnormal local MMP activity has been demonstrated in burn tissue [33,34], whether enzyme activity within actual burn wounds reaches the threshold required to activate these materials remains to be established. Infection-associated pH and ROS signals have similarly been exploited for the on-demand release of bioactive components [135], but direct validation in burn wounds remains limited.

By contrast, methacrylated hyaluronic acid (HAMA) microneedles carrying three-dimensional exosomes derived from human umbilical cord mesenchymal stem cells (hUCMSCs) have been investigated directly for deep partial-thickness burn repair [136]. This finding supports the feasibility of integrating localized delivery with regenerative therapy in burn wounds, but does not by itself establish microenvironment-triggered release. Local delivery and sustained delivery should therefore be distinguished from stimulus-responsive delivery, which requires a defined stimulus-dependent change in release behavior.

#### 4.3.3. Remodeling Phase: Long-Term Tissue Remodeling and Scar Outcomes

During the later stages of deep-burn healing, therapeutic priorities extend beyond wound closure toward collagen remodeling and the prevention or modulation of pathological scarring. Compared with the relatively extensive literature on early antibacterial and oxidative-stress regulation, direct evidence for responsive materials specifically targeting the remodeling phase of burn healing remains limited. MMP-responsive networks provide a conceptual strategy for coupling proteolytic signals to therapeutic release [70,101], but direct evidence demonstrating their activation during burn wound remodeling is still lacking.

AIE-based systems can enable pH visualization and have been used in animal studies that also assessed long-term scar outcomes [104]. However, monitoring wound-associated pH should not be interpreted as real-time monitoring of scar formation itself. Studies of scar-modulating and scarless-healing hydrogels [137,138] provide a broader design basis, but evaluation of responsive burn dressings will ultimately need to extend beyond short-term wound closure toward long-term tissue architecture and functional recovery.

From a materials-design perspective, several directions may warrant investigation for responsive hydrogels targeting the remodeling phase. One potential strategy is to regulate collagen metabolism by coupling MMP- or other protease-responsive structures to the local delivery of antifibrotic agents, such as imiquimod or 5-fluorouracil, or bioactive molecules that modulate TGF-β/Smad signaling [138]. Conceptually, such systems could use endogenous proteolytic activity to regulate local drug availability while reducing unnecessary systemic exposure. However, this represents a prospective design strategy rather than an established burn-responsive therapeutic mechanism, and would require direct validation of both the relevant enzymatic trigger and therapeutic response during burn remodeling.

A second direction involves adaptive mechanical regulation. Because mechanical stress is associated with hypertrophic scar development, hydrogels with temporally evolving mechanical properties could potentially reduce modulus or promote stress relaxation as remodeling progresses, thereby altering the mechanical environment experienced by myofibroblasts. Existing studies have explored relationships between hydrogel mechanics and scar formation [137,138], but responsive systems that directly couple burn microenvironmental signals to programmed mechanical changes remain insufficiently developed.

A third direction is long-term wound-state monitoring. Systems such as AIE-based pH visualization platforms [104] illustrate the feasibility of monitoring changes during wound healing. Future approaches could move beyond single-parameter pH sensing toward indicators more directly associated with remodeling status, including collagen deposition and vascular maturation, and ultimately integrate sensing outputs with therapeutic regulation. At present, however, these directions remain largely conceptual in the context of burn remodeling and should be regarded as priorities for future investigation rather than established therapeutic strategies.

Across these therapeutic phases, evaluation of responsive hydrogel systems should extend beyond gross wound closure. Inflammatory regulation should be assessed using cytokine profiles and macrophage polarization, whereas re-epithelialization should be evaluated based on epidermal continuity, keratinocyte proliferation, and restoration of barrier function. Angiogenesis should include both vessel density and vascular maturity, using markers such as CD31, α-SMA, and VEGF where appropriate. Long-term remodeling should be assessed through collagen organization, collagen I/III balance, α-SMA expression, TGF-β/Smad signaling, MMP/TIMP regulation, scar architecture, and tissue mechanics. Functional recovery may further require evaluation of tensile strength, elasticity, transepidermal water loss, tissue contraction, or joint mobility. Wound closure should therefore not be considered equivalent to complete tissue regeneration, because closure may result from wound contraction, mechanically induced hydrogel shrinkage, or scar formation rather than full re-epithelialization and functional recovery.

#### 4.3.4. Boundaries of Multifunctional Integration

Accordingly, increasing the number of integrated functions should not be equated with intelligent therapeutic regulation, which requires verifiable links among wound-state sensing, stimulus-dependent material behavior, and therapeutic output.

### 4.4. Temporally Programmed Responses and Intelligent Therapeutic Systems

The state of a burn wound and its therapeutic requirements evolve throughout the healing process. A key step in the further development of responsive materials is therefore to determine whether therapeutic outputs can be adjusted according to changes in wound status or treatment stage. Importantly, different levels of therapeutic control should be distinguished. A single-stimulus system establishes a relationship between one principal input and a material output; a multistimuli-responsive system incorporates multiple inputs; programmed therapy controls the sequence of multiple therapeutic events; and a closed-loop system uses sensed information to guide subsequent therapeutic decisions.

#### 4.4.1. Single-Stimulus Responsiveness

Single-stimulus systems use one principal input to regulate material behavior and therefore generally provide a relatively well-defined stimulus–response relationship. For example, H_2_O_2_ can induce cleavage of thioketal linkages in Cur@CMCTS-Tk and thereby promote drug release [82], whereas infection-associated acidification can trigger the release of TOB [109]. In contrast, Prussian blue nanozymes primarily regulate the wound microenvironment through ROS scavenging [86] and therefore should not be classified as a ROS-triggered network response.

Although a single input can establish a comparatively clear control pathway, no individual parameter such as pH, ROS, or temperature is sufficient to represent the overall state of a burn wound. The relevance of a single-stimulus system therefore continues to depend on whether its activation threshold and response direction correspond to the specific pathological conditions encountered in the target wound.

#### 4.4.2. Multistimuli Responsiveness

Multistimuli-responsive systems use two or more inputs to regulate material behavior. Dual- or multistimuli responsiveness should not automatically be interpreted as a molecular logic gate. A logic-gate behavior requires experimental evidence that multiple inputs jointly determine the material output according to a defined relationship. The HA/gelatin/nanozyme system exhibits dual pH/ROS responsiveness [90], while the PDMAEMA/HTCC system responds to both pH and temperature [74]. Nevertheless, the available evidence for these systems is insufficient to demonstrate that the two inputs jointly determine the output according to a defined Boolean relationship. Similarly, the antibacterial activity of QCS/NIPAM [100] and a dual-crosslinked network architecture [120] represent a therapeutic function and a network-construction strategy, respectively, rather than additional stimulus inputs.

More importantly, incorporating multiple inputs cannot compensate for a mismatch between the selected stimuli and the actual burn wound environment. Some pH/ROS-responsive systems were originally developed for acidic chronic wounds or diabetic infected wounds, with their response windows specifically tailored to acidic pathological conditions [119]. Likewise, a dual-drug EGCG–metformin system exhibiting pH/ROS responsiveness was evaluated in a diabetic infected wound model rather than an acute burn model [139]. Translation of these strategies to acute burns therefore requires reassessment of both the direction of the relevant stimuli and the corresponding activation thresholds.

#### 4.4.3. Programmed Therapy and Integrated Sensing–Therapeutic Systems

Programmed therapy focuses on controlling the sequence in which multiple therapeutic events occur. The Dual-Gel system [111], for example, combines bacteria-associated hyaluronidase-mediated nanovesicle release with light-mediated antibacterial treatment and sustained ROS regulation, thereby generating sequential therapeutic steps. A PNIPAM/SA bilayer dressing uses thermally induced mechanical contraction to promote wound-edge closure in deep partial-thickness burns [110]. pH-responsive bilayer systems can also enable sequential delivery of different therapeutic components [140]; however, the pH trajectory underlying such sequential behavior should not be assumed to represent the temporal evolution of acute burn wounds in general. Externally applied ultrasound provides another means of achieving on-demand or sequential release [54,55], although current examples primarily provide transferable mechanisms derived from non-burn settings.

A parallel direction involves systems capable of sensing wound status. AIE-based hydrogels can provide visual readouts of pH changes [104], whereas thermochromic patches can indicate changes in local temperature [106]. However, sensing capability alone does not constitute closed-loop therapy. A system that combines monitoring with externally initiated treatment still relies on an operator or external device to determine when therapeutic intervention should occur. A true closed-loop platform requires functional integration of sensing, decision-making, and therapeutic actuation, such that the detected wound state directly regulates the subsequent therapeutic output. Closed-loop platforms integrating sensing, control, and therapeutic modules have been explored in non-burn wound settings [141,142], supporting the feasibility of this system architecture rather than demonstrating that autonomous closed-loop regulation has already been achieved in burn-responsive hydrogels. Broader developments in intelligent wound monitoring and treatment have also been summarized in relevant reviews [143,144].

#### 4.4.4. From Preprogrammed to Adaptive Therapy

Temporally programmed therapy should also be distinguished from adaptive therapy. A preprogrammed multistage delivery system operates according to a predetermined material timescale, whereas a genuinely adaptive system must modify its therapeutic output according to the evolving state of the wound. This distinction is particularly relevant to burns because the severity of infection, duration of inflammation, and rate of tissue repair can vary considerably among wounds. Whether a fixed temporal program can accommodate such inter-wound variability therefore requires further validation.

Current intelligent wound-management systems also face challenges related to long-term stability, biofouling, sensor calibration, and quantitative readout [145]. For example, a system integrating a pH probe with ultrasound-responsive microcapsules can monitor wound status and subsequently release therapeutic agents upon externally applied ultrasound [146]. Nevertheless, this architecture integrates sensing with externally triggered therapy rather than providing autonomous closed-loop regulation.

Accordingly, the development of intelligent burn-responsive hydrogels should move beyond simply increasing the number of responsive modalities. A more meaningful progression is to establish reliable connections among wound sensing, state assessment, and therapeutic regulation, thereby shifting therapeutic control from predetermined material responses toward wound-state-driven intervention.

Representative control strategies and their evidence boundaries are summarized in Table 4.

## 5. Conclusions and Future Perspectives

### 5.1. Main Conclusions

Microenvironment-responsive hydrogels provide a promising design route for transforming burn wound dressings from passive coverings into materials capable of state-dependent regulation. Starting from the pathological characteristics of the burn wound microenvironment, this review conceptualizes responsive material design as a continuous process comprising pathological or external stimulus → responsive element → network transformation → therapeutic output. Current studies demonstrate that functionalization of natural and synthetic polymers, dynamic crosslinking, and multi-network architectures can enable inputs such as pH, ROS, proteases, temperature, externally applied light, and ultrasound to regulate network structure, cargo release, or therapeutic functions. At the same time, mechanical adaptability, wet-tissue adhesion, self-healing, and in situ gelation determine whether these responsive mechanisms can remain operational within the complex wound environment. By further integrating antibacterial activity, oxidative-stress regulation, and regenerative functions, responsive hydrogels are evolving from single-stimulus systems toward multistimuli responsiveness, programmed therapy, and integrated sensing–therapeutic platforms.

Overall, the central objective in developing microenvironment-responsive hydrogels is not to construct increasingly complex materials, but to improve the correspondence among burn-associated pathological signals, material response behavior, and therapeutic requirements. Translation from materials-level proof of concept toward clinical application will therefore depend on the simultaneous fulfillment of three conditions: reliable activation under authentic burn wound conditions, robust conversion of network-level changes into therapeutic outputs, and systematic validation of safety, manufacturability, and long-term therapeutic outcomes.

### 5.2. Current Challenges and Future Directions

The current evidence base should be interpreted at three distinct levels: (i) demonstration of a defined stimulus-responsive mechanism at the material level, (ii) demonstration of therapeutic efficacy in a burn model, and (iii) evidence that the corresponding signal within an actual burn wound can trigger the intended material response in situ. These levels of evidence are related but should not be considered interchangeable. Although some studies have demonstrated both material responsiveness and therapeutic efficacy in burn models, whether the intensity, spatial distribution, and temporal evolution of stimuli within actual wounds are sufficient to reach the activation thresholds of the materials often remains inadequately established. This limitation is particularly relevant to endogenous signals such as pH, ROS, and proteases, which exhibit substantial temporal dynamics and spatial heterogeneity, and to responsive systems originally developed for diabetic wounds, chronically infected wounds, or other pathological conditions. Future studies should therefore progress from simply demonstrating that a material can respond toward establishing that it responds appropriately within the target burn environment. Analysis of patient-derived wound fluid, longitudinal characterization of the wound microenvironment, and quantitative determination of material activation thresholds could help establish more direct relationships between pathological signal ranges and responsive chemistries.

Evaluation frameworks should likewise extend beyond short-term wound closure toward a more complete mechanistic and therapeutic evidence chain. Current studies rely extensively on in vitro experiments and animal burn models, although differences in skin architecture and wound-healing processes can limit direct extrapolation to humans [148]. For responsive materials, reporting an in vitro response profile, antibacterial efficacy, or wound-closure rate in an animal model alone is insufficient to establish overall therapeutic relevance. Future evaluation should incorporate models that more closely reproduce human burn pathophysiology and examine whether material responsiveness remains robust under conditions more representative of burn wounds, including high water content, ionic strength, protein abundance, and enzymatic activity. Long-term tissue remodeling, scar formation, and functional recovery should also be incorporated into outcome assessment. Meanwhile, mechanistic evidence derived from non-burn models should be accompanied by explicit consideration of its range of transferability, rather than treating material-level feasibility as direct evidence of therapeutic effectiveness in burns.

The balance between material complexity and clinical translatability represents another important challenge. Sterilization by gamma irradiation, electron-beam irradiation, ethylene oxide, or aseptic processing may affect dynamic covalent bonds, ROS-sensitive linkers, photoinitiators, nanoparticles, therapeutic proteins, and drug-release behavior. Therefore, sterilization validation should include not only sterility testing but also post-sterilization evaluation of swelling, mechanical properties, activation thresholds, degradation, and release kinetics. Storage stability should be assessed with respect to water loss, premature cargo release, oxidation of responsive groups, nanoparticle aggregation, and changes in gelation or phase-transition behavior. Scale-up may introduce batch-to-batch variations in polymer molecular weight, degree of substitution, crosslinking density, nanomaterial dispersion, and gelation time. Accordingly, responsive hydrogels should be controlled using predefined critical quality attributes, including composition, swelling ratio, modulus, adhesion, stimulus-activation threshold, release profile, degradation rate, and sterility. Material design should therefore prioritize a limited number of mechanistically defined functions rather than continuously increasing the number of responsive modalities or therapeutic components. Irradiation-related processing should be distinguished from externally triggered photothermal or photoresponsive therapy. Ultraviolet or visible-light irradiation may be used for photochemical crosslinking, whereas gamma irradiation and electron-beam irradiation can be used for network formation, grafting, or terminal sterilization [149]. Because sterilization may alter the structure and functional performance of hydrogels, the effects of the selected process on network integrity, swelling behavior, mechanical properties, drug retention, and cargo bioactivity should be evaluated separately.

A longer-term objective is to enable therapeutic output to adapt dynamically to the evolving state of the wound. Burn-related studies have already explored stimulus responsiveness, programmed release, and wound-state monitoring; however, based on the available evidence, most systems have not yet achieved fully autonomous closed-loop regulation. Device-level closed-loop platforms developed for non-burn wounds provide transferable system-level principles for integrating sensing, analysis/control, and on-demand therapy [142,147], but do not constitute direct evidence that equivalent autonomous regulation has been achieved in burn-responsive hydrogels. Accordingly, the long-term development of intelligent burn hydrogels should extend beyond the addition of responsive moieties, therapeutic components, or sensing modules. The more substantive goal is to establish a reliable continuum of wound sensing → state assessment → therapeutic regulation → outcome feedback, and ultimately to determine whether this control process remains stable and therapeutically meaningful within the dynamic and heterogeneous burn wound microenvironment.

## Figures and Tables

**Table 1:** Major stimuli relevant to microenvironment-responsive hydrogel design for burn wounds.

Stimulus	Evidence/Relevance in Burn Wounds	Representative Responsive Mechanism	Key Design Consideration	Ref.
pH	Acute burn wounds are generally alkaline, although pH varies with healing stage, infection, burn depth, and measurement conditions.	Protonation/deprotonation; cleavage or exchange of pH-sensitive bonds	Match the response direction and activation threshold to burn-relevant pH ranges.	[10,11,13,32]
ROS	Oxidative stress is increased after burns, whereas absolute local ROS concentrations remain insufficiently quantified in human wounds.	Oxidation or cleavage of ROS-sensitive groups, e.g., thioketal and boronate ester	Do not directly equate in vitro H_2_O_2_ concentrations with ROS levels in burn wounds.	[14,46]
MMPs and other proteases	Increased MMP activity has been detected in burn tissue and biological fluids.	Enzymatic cleavage of peptide crosslinks or degradable substrates	Distinguish systemic biomarkers from local enzymatic activity and validate cleavage thresholds.	[16,33,34]
Temperature	Can regulate thermoresponsive polymers but is not a burn-specific pathological signal.	Thermally induced phase transition or network contraction	Best considered a physiological or externally controllable trigger rather than a burn biomarker.	[56,58]
NIR	Externally controllable and applicable to photothermal/photodynamic treatment.	Photothermal conversion or photosensitization	Report wavelength, power density, and irradiation duration.	[60]
Ultrasound	Can regulate network behavior or therapeutic release.	Cavitation, mechanical effects, piezoelectric/sonodynamic activation, or triggered release	Distinguish material-mediated responses from the direct biological effects of ultrasound.	[51,52,53,54,55]

**Table 2:** Characterization methods and key quantitative readouts for stimulus-responsive hydrogels in burn-wound applications.

Stimulus-Responsive Behavior Assessed	Characterization Method	Key Quantitative Readouts
pH-responsive swelling/deswelling	Swelling and deswelling tests under wound-relevant pH conditions	Equilibrium swelling ratio, stimulus-induced change in swelling, response/recovery time, and cyclic reversibility
ROS- or enzyme-responsive degradation	Incubation in stimulus-containing and matched control media, followed by degradation analysis	Remaining mass, degradation rate, structural integrity, modulus retention, and response time
Stimulus-triggered cargo release	In vitro release tests under stimulus and matched control conditions	Cumulative release, release rate, burst-release fraction, release kinetics, and stimulus selectivity
Thermoresponsive phase transition	Temperature-dependent turbidity or transmittance measurements	Transmittance/turbidity change, transition temperature (transition temperature or LCST), and reversibility during heating–cooling cycles
Thermoresponsive sol–gel transition	Temperature-sweep oscillatory rheology	Storage modulus (*G*′), loss modulus (*G*″), gelation temperature, and temperature-dependent viscoelasticity
Externally triggered response, where applicable	Calibrated light/NIR or ultrasound stimulation with sham controls	Stimulus dose, local temperature or acoustic output, changes in material properties or cargo release, response time, and cycle retention
Stimulus-induced surface wettability, where applicable	Water contact-angle measurements before and after stimulation	Water contact angle, stimulus-induced change in contact angle (Δθ), and reversibility

Notes: A stimulus-responsive claim should be supported by comparisons with matched control conditions. Surface wettability should be described as stimulus-induced only when a reproducible stimulus-dependent change is demonstrated.

**Table 3:** Representative responsive hydrogel systems for burn wound treatment.

Hydrogel System	Stimulus or Trigger	Material Response	In Vivo Model and Therapeutic Outcome	Ref.
Cur@CMCTS-Tk	50 mM H_2_O_2_	Curcumin release: 53.4 ± 3.9% at 5 h; WVTR increased from 1376 to 2381 g·m^−2^·d^−1^ during 14 d H_2_O_2_ exposure	Full-thickness burn rat model; wound closure of approximately on day 14	[82]
CS/AST/IBU	Physiological temperature, 37 °C	Gelation time approximately 800 s; IBU and AST release approximately 70% and 55% within 24 h, respectively	High-voltage, low-current electrical burn rat model; significantly reduced residual wound area on day 5, although the exact percentage was not reported	[83]
CCS/BGM	0.3% H_2_O_2_; 405 nm irradiation for 1 min	Swelling ratio approximately 25.9%; degradation approximately 25.7% after 3 d	Combined radiation–burn mouse model; wound closure reached 96.82% on day 14 and 100% on day 21	[91]
CAP hydrogel	pH 5.0 or 10 mM H_2_O_2_	Carnosic acid release reached 82.48% at pH 5.0 and 84.8% under 10 mM H_2_O_2_ after 120 h	Deep-burn mouse model; wound closure approximately 93% on day 20	[95]
CMCS/PUE double network	pH 5.5 and pH 7.4	PUE release at pH 7.4 was approximately 20% higher than at pH 5.5; bacterial survival rates were 7.17% for *S. aureus* and 4.37% for *E. coli*	*S. aureus*-infected burn rat model; residual wound area was 49.83%, 28.50%, and 11.97% on days 7, 10, and 13, respectively	[108]
QCS/OD/TOB/PPY@PDA	Bacterial acidification and NIR irradiation	Bacteria-responsive tobramycin release; bactericidal activity sustained for approximately 11 d	*P. aeruginosa*-infected burn model; improved bacterial clearance, inflammation control, collagen deposition, vascularization, and wound closure; the exact wound-closure percentage was not reported in the cited study	[109]
GPC-BTC	pH 4.5–6.0; 808 nm NIR irradiation	Cu^2+^ release at pH 5.0: 54.9% at 24 h; photothermal conversion efficiency of BTC solution: 29.3%; antibacterial inhibition under NIR irradiation approximately 100%	Full-thickness infected burn mouse model; complete bacterial clearance after 3 d; the exact wound-closure percentage was not reported in the cited study	[105]
PVA/BBR/HD	pH 5–8	Visible-mode detection limit: 0.3 pH units; response time approximately 1 min; BBR release: 26.75 ± 0.93% at 24 h; smartphone-based readout within 1 s	Second-degree burn model; near-complete wound healing on day 14	[104]
PNIPAM/SA bilayer	Thermal stimulation	Young’s modulus: 9.9–16.7 kPa; WVTR approximately 2500 g·m^−2^·d^−1^ for the inner layer and 1500 g·m^−2^·d^−1^ for the outer layer	Burn wound model; wound-healing rate approximately 92% on day 21	[110]
Dual-Gel	Hyaluronidase + light	Hyaluronidase-responsive nanovesicle release followed by light-induced ROS generation; the outer layer continuously consumes excess ROS	Infected burn model; residual wound area approximately 3.8% on day 14, corresponding to approximately 96.2% wound closure	[111]

Notes: NR, not reported in the cited study. Because different studies used different burn models, observation time points, and definitions of wound healing, direct comparison among hydrogel systems should be made cautiously.

**Table 4:** Representative control strategies toward intelligent and adaptive wound therapy.

Control Level	Representative System	Input	Control Principle	Evidence Boundary	Ref.
Multistimuli response	HA/Gelatin nanozyme	pH + ROS	Two pathological inputs regulate network/release behavior	Non-burn; no demonstrated Boolean logic	[90]
Multistimuli response	PDMAEMA/HTCC	pH + temperature	Combined pH-responsive and temperature-dependent behavior	Material-level evidence; no burn validation	[74]
Programmed therapy	Dual-Gel	Hyal + light	Sequential release: antibacterial treatment followed by ROS regulation	Burn model	[111]
Programmed therapy	bFGF-CFMN bilayer	Stage-dependent pH	Sequential therapeutic delivery	Burn/frostbite model; prescribed pH trajectory should not be generalized	[140]
Externally controlled therapy	Ultrasound scaffold	Ultrasound	On-demand/sequential release	Non-burn transferable mechanism	[54]
Closed-loop therapy	SWPS	Bacterial DNase	Infection sensing → analysis/control → electrical stimulation	Non-burn device-level closed loop	[147]
Closed-loop therapy	IWP	Wound pH	Sensing → analysis/control → on-demand therapeutic delivery	Non-burn device-level closed loop	[142]
Sensing + external actuation	pH probe/US hydrogel	pH + ultrasound	Monitoring followed by externally triggered release	Non-burn; not autonomous closed loop	[146]

Notes: Arrows represent the sequential working flow of the therapeutic system: from infection sensing, to analysis and control, and finally to electrical stimulation output.

## Data Availability

No new data were created or analyzed in this study. Data sharing is not applicable to this article.
